# Know your full potential: Quantitative Kelvin probe force microscopy on nanoscale electrical devices

**DOI:** 10.3762/bjnano.9.172

**Published:** 2018-06-15

**Authors:** Amelie Axt, Ilka M Hermes, Victor W Bergmann, Niklas Tausendpfund, Stefan A L Weber

**Affiliations:** 1 Max-Planck-Institute for Polymer Research, Ackermannweg 10, 55128 Mainz, Germany; 2 Institute of Physics, Johannes Gutenberg University Mainz, 55099 Mainz, Germany

**Keywords:** AM-KPFM, AM lift mode, AM off resonance, AM second eigenmode, cross section, crosstalk, field effect transistor, FM-KPFM, frequency modulation heterodyne, frequency modulation sideband, quantitative Kelvin probe force microscopy, solar cells

## Abstract

In this study we investigate the influence of the operation method in Kelvin probe force microscopy (KPFM) on the measured potential distribution. KPFM is widely used to map the nanoscale potential distribution in operating devices, e.g., in thin film transistors or on cross sections of functional solar cells. Quantitative surface potential measurements are crucial for understanding the operation principles of functional nanostructures in these electronic devices. Nevertheless, KPFM is prone to certain imaging artifacts, such as crosstalk from topography or stray electric fields. Here, we compare different amplitude modulation (AM) and frequency modulation (FM) KPFM methods on a reference structure consisting of an interdigitated electrode array. This structure mimics the sample geometry in device measurements, e.g., on thin film transistors or on solar cell cross sections. In particular, we investigate how quantitative different KPFM methods can measure a predefined externally applied voltage difference between the electrodes. We found that generally, FM-KPFM methods provide more quantitative results that are less affected by the presence of stray electric fields compared to AM-KPFM methods.

## Introduction

In this study, we compare the most commonly used amplitude modulation (AM) and frequency modulation (FM) Kelvin probe force microscopy (KPFM) methods under ambient conditions to investigate how these methods can measure quantitative variations in the local contact potential difference (CPD). KPFM is a scanning force microsopcy (SFM) method that correlates the local electric potential landscape with local topographic information. Thus, KPFM is ideally suited to characterize of a variety of nanostructured semiconducting systems such as electronic devices [1] and solar cells [2].

To understand and improve the charge carrier generation and extraction within a solar cell, the local potential distribution needs to be correlated to the constituent layers of the cell. Therefore, a high lateral resolution together with a reliable quantification of the local potential is required. In the past, KPFM measurements have frequently been used to image potential distributions on cross sections of a range of different solar cell devices, including organic [3–5], and inorganic [6] as well as hybrid perovskite solar cells [7–15].

**Figure 1:**
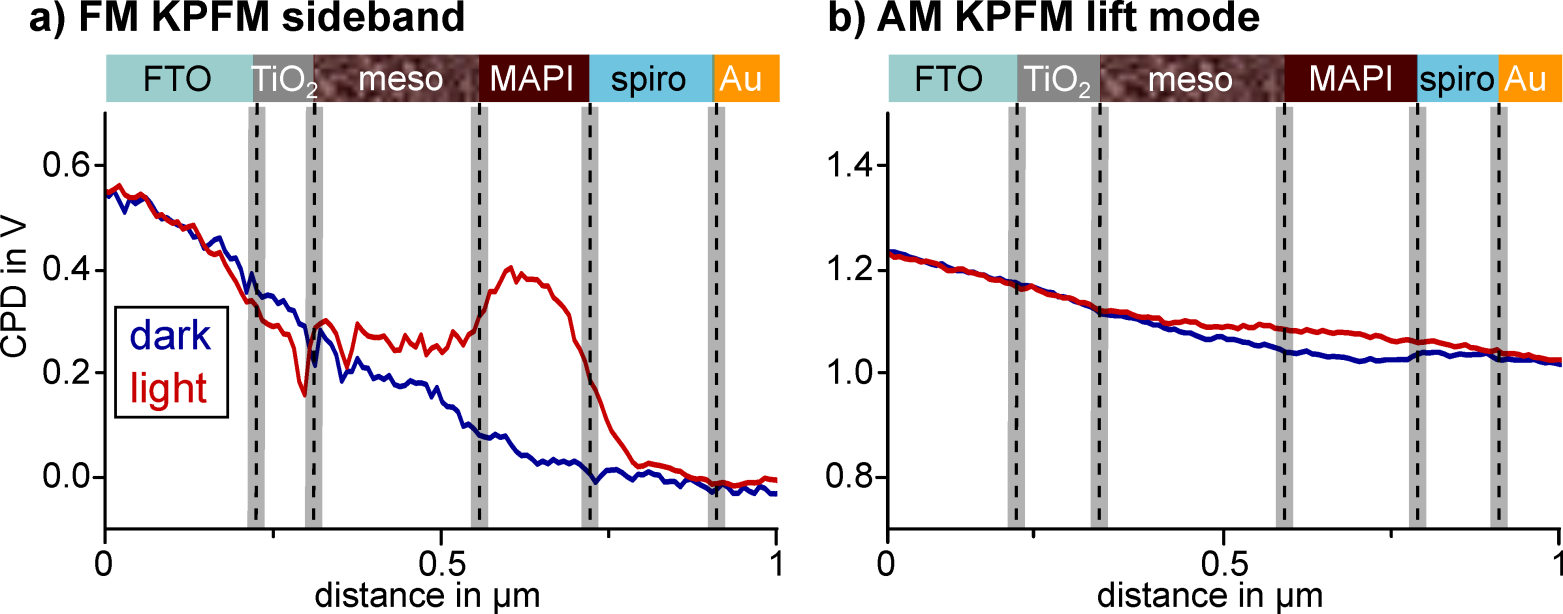
CPD line profiles of two KPFM experiments on the same cross section of a mesoscopic perovskite solar cell under short circuit conditions with and without illumination, visualized by the red and blue line, respectively. The cell consisted of a fluorine-doped tin oxide (FTO) electrode, a compact TiO_2_ electron extraction layer and a mesoscopic TiO_2_ layer (meso) filled with the perovskite light-absorber methylammonium lead iodide (MAPI). The mesoscopic layer was followed by a compact MAPI capping layer, the hole transport material spiro-OMETAD and a gold electrode. Prior to the measurement the cross section of the solar cell was polished with a focused ion beam (FIB) to minimize topographic crosstalk. The CPD line profiles in a) were extracted from double side band frequency modulation KPFM (FM sideband) scans in single pass with VAC of 3 V [7]. The CPD line profiles in b) were extracted from amplitude modulation KPFM (AM lift mode) scans in lift mode with a tip–sample distance of 10 nm, an oscillation amplitude of ≈80 nm and a tip voltage *U*_AC_ of 1 V. Each line profile is an average of three adjacent scan lines.

In the course of one of our KPFM studies on a cross section of a perovskite solar cell under operating conditions [7] we observed fundamental differences in the potential distribution when using FM sideband KPFM as compared to AM lift mode KPFM (Figure 1). The cell was under short circuit conditions and could be illuminated with a white light source from the side. Further details on the solar cell, the sample preparation and experimental setup are given in the figure caption and in [7].

The FM- and AM-KPFM data was collected in subsequent measurements with the same cantilever on the same solar cell cross section. However, the resolved potential distributions differed significantly. In dark, the potential drop from FTO to gold measured with FM-KPFM was around −0.55 V, while the potential difference between the electrodes detected with AM-KPFM was only −0.25 V. Furthermore, the absolute potential detected in AM-KPFM had an offset of +1 V. The most fundamental difference in the potential distributions imaged in FM- and AM-KPFM could be observed upon illuminating the sample. While FM-KPFM resolved a +0.35 V increase of the potential within the methylammonium lead iodide (MAPI) capping layer as well as a narrow local minimum featured at the interface of the compact and the mesoscopic TiO_2_ layer, AM-KPFM detected only a slightly higher potential in the mesoscopic TiO_2_ and the MAPI capping layer. The illumination-induced potential difference resolved by AM-KPFM was less than 50 mV and no local features could be observed. Thus, only using AM lift mode, we likely would have missed the illumination induced changes in the potential distribution, which we assigned to unbalanced charge extraction from the perovskite layer. The absence of local features in the potential distribution imaged with AM lift mode KPFM, the potential offset of +1 V, as well as the reduced potential increase upon illumination suggested that the spatial and quantitative resolution of AM lift mode KPFM was not sufficient to characterize the potential distribution within the solar cell.

For future studies it is therefore important to know the limitations of different KPFM techniques to characterize samples most efficiently by choosing an appropriate operation mode. Since the invention of KPFM, a vast number of studies have investigated differences in lateral and voltage resolution of AM and FM methods. Polak et al. have investigated, how AC coupling between excitation and cantilever deflection signal affects the measured potentials in AM-KPFM [16]. Generally, FM-KPFM is less affected by AC crosstalk artefacts, as excitation and detection are performed at different frequencies. Other influences that have been investigated were the cantilever orientation with respect to a structured sample [17], the tip–sample distance [17–20], topographic or capacitive cross talk [19,21–22] and the choice of frequencies. All in all, an overwhelming number of studies have reported a superior lateral resolution, both laterally and in voltage, for FM-KPFM [18–19,23–26]. Li et al. [19] also reported a higher sensitivity for the potential detection.

Until now, most studies comparing AM- to FM-KPFM methods have used samples exhibiting a work function contrast [23–24,27]. For example, Zerweck et al. have observed a superior spatial and quantitative resolution of FM-KPFM as compared to AM-KPFM on a gold and potassium chloride interface in ultra-high vacuum [23]. However, such measurements are difficult to interpret as it is unclear what the expected workfunction contrast is. This is particularly important for measurements under ambient conditions, where adsorption layers can distort the CPD contrast [28].

In our study we follow an idea by Ziegler et al., who investigated the potential resolution of lift mode AM- and FM-KPFM in air [18]. The authors used a microscopic electrode that was set on a defined external bias. Furthermore, the influence of stray electric fields on the measured potential was investigated by varying the background voltage on the silicon substrate. Here, we use an array of micron-scale interdigitated electrodes on glas with a defined potential difference applied between neighboring electrodes. By investigating the pre-defined potential difference between the electrodes, a possible influence of tip- or sample contamination can be minimized. We furthermore investigate the influence of stray electric fields by adding a metal electrode underneath the sample.

The goal of this work is to complement the previous comparative KPFM studies by a comprehensive investigation on the reliability of the potential mapping of five common KPFM techniques under ambient conditions. We compare AM-KPFM in lift mode, on the second eigenmode and off resonance, as well as FM-KPFM with double sideband detection and heterodyne FM-KPFM.

## Theory

KPFM [29] utilizes a conductive SFM tip as Kelvin probe [30] to map electrical surface potential variations on a nanometer scale [31]. To quantify the potential difference between the tip and a sample, the electrostatic field is enhanced by additionally applying a voltage between tip and sample. In electrostatic force microscopy [32], an alternating voltage *U*_AC_ is applied and the response tracked by means of a lock-in amplifier. Thereby, two different detection methods can be used: The amplitude modulation (AM) mode tracks variations in the response amplitude, whereas frequency modulation (FM) mode tracks variations in the cantilever’s resonance frequency, e.g., via the phase lag between excitation and response. By applying an additional DC voltage *U*_DC_ to the tip, the electrostatic force is minimized if *U*_DC_ = *U*_CPD_, where *U*_CPD_ is the contact potential difference between the tip and the sample. This is the basic operation principle of KPFM [31]. This section will introduce the operation principles of the AM- and FM-KPFM detection modes and discuss possible benefits and drawbacks.

Generally, *U*_CPD_ describes the difference in the Fermi levels


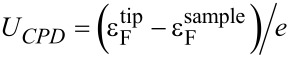


of tip and sample (*e*: elemental charge), which also contains information about an externally applied bias [18], static charges [27] , or local electronic excitations [33]. In equilibrium, *U*_CPD_ corresponds to the difference in work functions of the tip and the sample material.

We can calculate the electrostatic force on a SFM tip by considering the capacitance *C* of the gap between cantilever/tip and the sample. From the capacitor’s energy *W* = 1/2 *C* (Δ*U*)^2^, we can derive the electrostatic force as

[1]
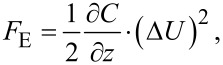


with the tip–sample distance *z* and the potential difference between the tip and the sample Δ*U* = *U*_ext_ − *U*_CPD_. Here, *U*_ext_ is the sum of all externally applied voltages to tip or sample. If we keep the sample grounded and apply an external voltage to the tip with both an alternating AC voltage and a constant bias in the form *U*_ext_ = *U*_DC_ + *U*_AC_ sin(ω_E_*t*), the resulting electrostatic force can be divided into one static and two dynamic spectral components [34]:

[2]
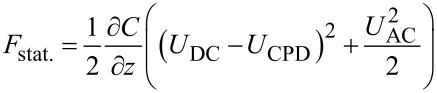


[3]
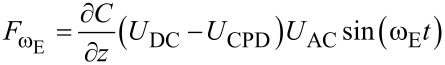


[4]
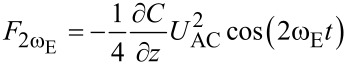


Equation 3 is the fundamental equation describing AM-KPFM: When *U*_DC_ = *U*_CPD_, the amplitude of the response at the angular frequency ω_E_ vanishes. In AM-KPFM, a feedback loop that minimizes the response amplitude by adjusting *U*_DC_. AM detection is ususally more prone to artifacts such cross coupling of the AC drive signal, e.g., into the shaker piezo [21]. Furthermore, Equation 3 shows that the amplitude of the electrostatic force is proportional to the gradient in capacitance. Colchero et al. have shown that for most common tip/cantilever geometries the large surface area of the tip cone and the cantilever yields a significant contribution to the gradient in capacitance, even at tip–sample distances of only a few nanometers [20]. This so-called stray capacitance [35] can decrease the lateral resolution by averaging the surface potential over a larger area.

To reduce the effect of the long-ranged electrostatic interaction of the cantilever, force gradient detection can be used [18,20,23]. The presence of a tip–sample force field *F*_ts_(*z*) causes a shift in the angular resonance frequency ω_0_ of the cantilever. For small oscillation amplitudes, the modified angular resonance frequency 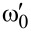 can approximately be described by means of an effective spring constant


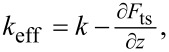


where *k* is the undisturbed spring constant of the cantilever:

[5]
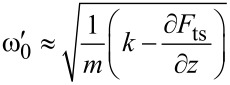


Thus, an alternating voltage *U*_AC_ not only causes periodic fluctuations in the electrostatic force (Equation 3), but also in the resonance frequency. The magnitude of this frequency modulation is proportional to the electrostatic force gradient and thereby to the second derivative ∂^2^*C*/∂*z*^2^ = *C*′′ of the capacitance. Thus, FM detection is more sensitive to the electrostatic interaction of the tip apex with the sample surface [20].

Originally, the peroiodic oscillations in Δ*f* were directly detected by means of a phased-locked loop in non-contact AFM under ultrahigh vacuum conditions. An elegant way of detecting the electrostatic frequency modulation is to use non-linear frequency mixing with a mechanical cantilever oscillation at angular frequency ω_m_, such as the tapping oscillation used for the height feedback [36]. As the capacitance gradient monotonically decreases away from the surface, it will also oscillate with frequency ω_m_. Thus, the capacitance gradient can be written as a Fourier series


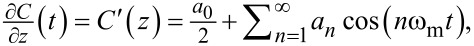


and the electrostatic force (Equation 1) can be written as:

[6]
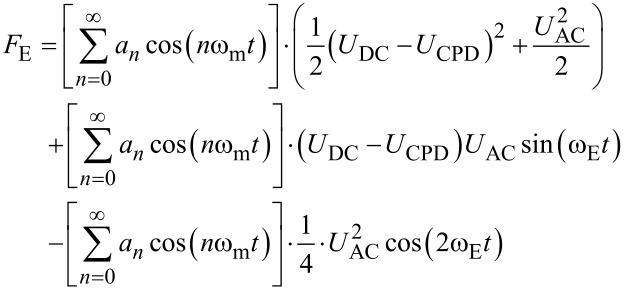


By only considering Fourier coefficients up to *n* = 1, we can again calculate and separate different spectral components of the electrostatic force:

[7]
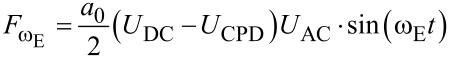


[8]
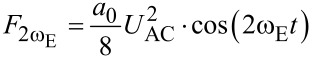


[9]
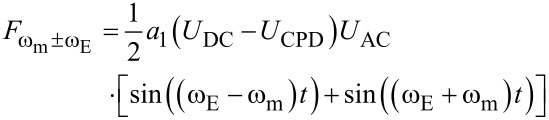


[10]
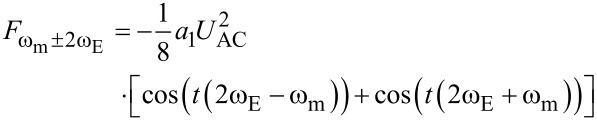


Equation 7 and Equation 8 are equivalent to the AM-KPFM-equations Equation 3 and Equation 4. We can find the connection between the Fourier coefficient and the capacitance gradient as *a*_0_ = 2*C*′ (See Supporting Information File 1 for a formal proof). Equation 9 and Equation 10 show that electrostatic signals can also be detected at the sidebands 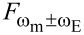 and 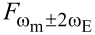 of the mechanical oscillation at ω_m_. In particular, Equation 9 also contains a factor (*U*_DC_ − *U*_CPD_) in analogy to Equation 7. This is the fundamental equation describing FM-KPFM. In the Appendix we show that the Fourier coefficient *a*_1_ is proportional to the mechanical oscillation amplitude and to *C*′′.

As *C*′′ is more sensitive to local tip–apex/sample interactions [20], FM-KPFM usually leads to a superior lateral and voltage resolution [18–19,23–26]. On the other hand, the force signal is usually much stronger than the force gradient signal. Thus, higher electrical drive amplitudes are usually required for FM-KPFM that can cause other problems, such as band bending [37].

## Methods

**Figure 2:**
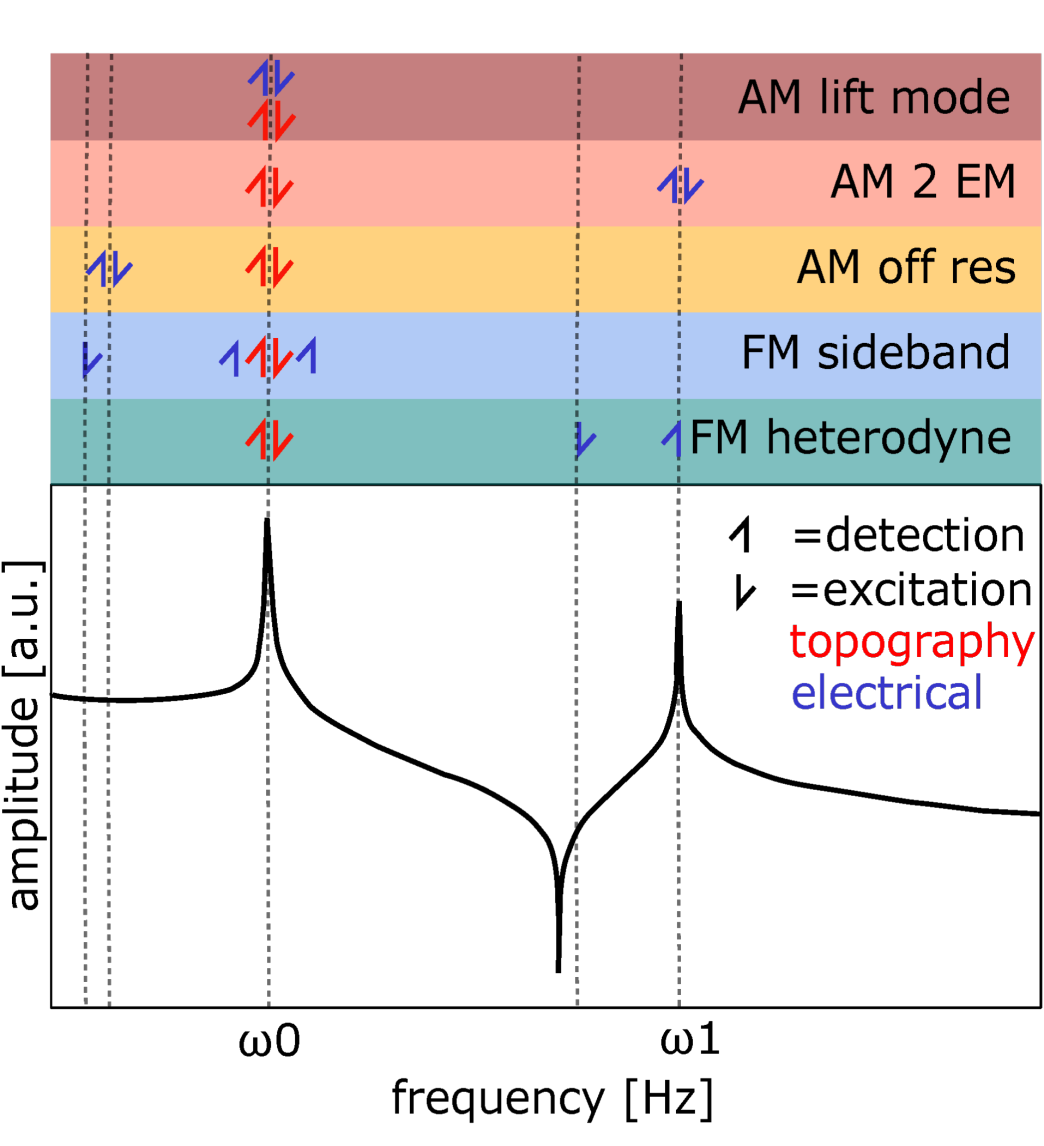
Overview of excitation and detection frequencies for KPFM methods used in this work. The lower part shows the transfer function of the cantilever, amplitude plotted vs frequency. The upper part shows excitation (arrow upwards) and detection (arrow downwards) for the corresponding methods with the respective frequencies. Red color is used for the topography signal and blue for the electrical excitation and detection. The color code in the upper part corresponds to plots in the results. Representation inspired by [26].

An overview and simplified representation of all the KPFM methods used in this study are given in figure Figure 2. AM-KPFM is the most commonly used method on most commercial scanning probe microscopy systems, mainly due to its easy implementation. Nevertheless, there are different ways to operate AM-KPFM. In the simplest form, an AC voltage is applied during normal tapping mode imaging (single scan) at a frequency far below the first resonance ω_E_ *<<* ω_0_. We refer to this mode as AM-KPFM off resonance (AM off res). This mode is implemented on older AFM systems, where the auxiliary lock-ins were limited in terms of the maximum frequency they could measure. The biggest drawback of this method is the lower signal-to-noise ratio (SNR) resulting from the off-resonance detection. The SNR can be improved by choosing an ω_E_ at one of the cantilever’s eigenmodes. We refer to this mode as AM-KPFM second eigenmode (AM 2 EM), where the topography is measured at the first, and the CPD is measured on the second eigenmode. Finally, in AM-KPFM lift mode (AM Lift mode) the topography and CPD measurements are decoupled: In a first step, a topographic contour line is recorded in tapping mode. In a second step, the mechanical excitation is switched off and the tip follows the same contour line shifted in *z*-direction by a defined lift height, typically 10–100 nm above the sample. AM-KPFM in lift mode has the advantage that in theory the electrostatic response is completely decoupled from any other short-ranged forces that act on the tip during the tapping motion. Furthermore, detecting at the first eigenmode results in an improved SNR. At the same time, the larger tip–sample distance reduces the lateral resolution and the image acquisition time is a factor of two longer, since every line needs to be scanned twice.

In FM-KPFM, the force gradient-sensitive sidebands introduced in Equation 9 are used to measure the CPD. In the mode that we refer to as FM Sideband KPFM, the frequency of electrical excitation is lower than the first resonance ω_E_ *<<* ω_0_ while the detection is performed at ω_m_ ± ω_E_ with the mechanical oscillation frequency ω_m_ at the first resonance. To decouple the detection of the sidebands from the mechanical carrier signal, ω_m_ should be sufficiently high. Nevertheless, choosing ω_m_ too high shifts the sidebands further away from the resonance frequency, decreasing the SNR. Thus, FM sideband KPFM typically has to be performed at higher AC voltages and/or at low detection bandwidths, limiting the speed of the measurement. In FM Heterodyne KPFM [26,38], the electrical excitation is performed at ω_E_ = ω_1_ − ω_0_, which shifts the sideband frequency to the second eigenmode at ω_1_. Here, the big advantage is that resonance amplifies the response without limiting the detection bandwidth, providing an improved SNR and faster imaging speeds [26].

## Experimental

We used an Asylum research MFP3D SFM in a nitrogen glovebox (level of humidity below 1%) for all experiments. The typical resonance frequency of the cantilevers (Bruker Model:SCM-PIT-V2) was ≈75 kHz, spring constant of 3 N/m, a tip radius of 25 nm and a tip height of 10 to 15 μm. The typical length of the cantilevers was ≈225 μm, the width ≈35 μm. Tip, tip cone and cantilever are coated with PtIr (work function 5.5 eV [39]) on both sides. The topography feedback was performed with amplitude modulation (AM) on the first eigenmode and the oscillation amplitude was kept to approximately 40 nm for all methods. To perform the KPFM feedback, we used a Zurich Instruments HF2LI for all methods except for AM liftmode, where we used the implementation of the Asylum system (NAP mode). On the Asylum system, the CPD signal recorded during the nap scan is applied to the tip during the topography scan. Thereby, electrostatic tip–sample interaction is minimized (Feed-forward compensation [40]). The lift height was set to 10 nm. As we show in the wiring scheme (Figure S14 and Figure S15, Supporting Information File 1) *U*_DC_ is applied to the tip. We connected the cantilever chip with an external wire to minimizie electrical crosstalk like reported by Polak et al. [16]. The parameters of the measurements can be found in Table 1. The feedback was optimized for 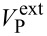 = 0.5 V. The test structure was based on an interdigitated electrode array (IDA-Pt 2 μm by ALS, Japan). The pitch of the 90 nm thick platinum electrodes is 4 μm, the width of the electrodes is 2 μm and the length 2 mm. The electrode array consists of 65 pairs of electrodes. According to ALS-Japan the electrodes are made of pure platinum with a work function of 5.7 eV [41]. The electrodes are embedded into the glass substrate and therefore offer a low resistance while keeping the topography variations below ≈50 nm, minimizing the effect of topographic cross talk. To further eliminate the influence of variations in the work function between the electrodes and on the tip, we applied a defined voltage between the electrodes and only considered the potential difference between neighboring electrodes. Thus, the measured potential difference is not influenced by contamination of the surface, the tip or local changes in the materials. To minimize the influence of potential variations along the electrodes we furthermore disabled the slow scan axis during the measurements. To study the effect of stray fields, aluminum foil was placed under the substrate of the electrode array, where we could apply an external voltage of 200 V. To show that KPFM operates in a linear regime (Equation 11) we recorded bias spectroscopy sweeps prior to every measurement (Figure S1 and Figure S2, Supporting Information File 1).

**Table 1:** Overview of methods and parameters, topography carrier in all cases is the first eigenmode of the cantilever ω_t_ = ω_0_. ω_1_ represents the second eigenmode of the cantilever.

Method	electrical excitation	electrical detection	feedback	*U*_AC_

AM Lift Mode	ω_E_ = ω_0_	ω_0_	*X*(ω_0_) = min	1 V
AM 2 EM	ω_E_ = ω_1_	ω_1_	*X*(ω_1_) = min	1 V
AM Off Res	ω_0_*>>* ω_E_ = 10 kHz	ω_E_	*X*(ω_E_) = min	1 V
FM Sideband	ω_0_ *>>* ω_E_ = 1.5 kHz	ω_0_ ± ω_E_	*X*(ω_0_ + ω_E_) − *X*(ω_0_ − ω_E_)	2 V
FM Heterodyne	ω_E_ = ω_1_ − ω_0_	ω_1_	*X*(ω_1_) = min	1 V

## Results and Discussion

**Figure 3:**
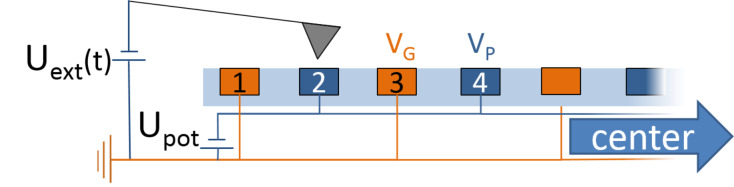
Sketch of the setup as well as marking scheme of electrodes on the edge. *U*_ext_ represents the electrical excitation applied to the cantilever, *U*_pot_ the potential applied to the electrodes.

**Figure 4:**
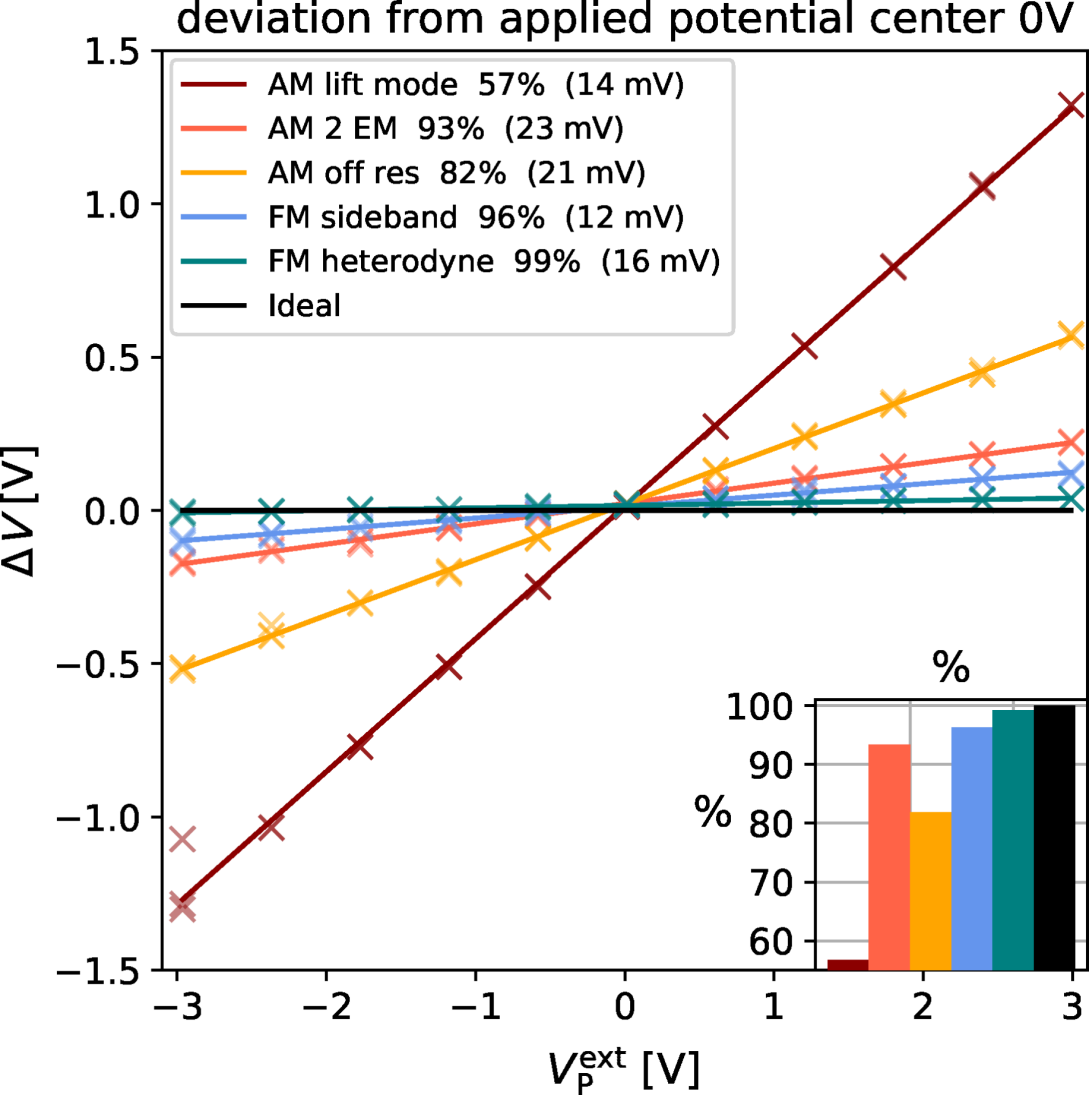
Comparison of the deviation of the measured potential difference from the applied potential plotted against the applied potential for AM (warm colors) and FM (cool colors), black represents an ideal measurement. Inset in the lower right visualizes the fraction of the potential captured by the respective method. Legend in the upper left shows the offset in brackets. Data shown captured in the middle of the electrode structure without an additional electrostatic force.

We performed the first measurement in the center of the electrode structure (Figure 3) at a position, where the entire length of the cantilever was positioned over the electrode array. The cantilever was placed perpendicular to the electode stripes. This arrangement mimics both the stray field and the potential distribution of flat electronic devices like field effect transistors. Every second electrode on the interdigitated array was grounded (Figure 3) 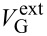 = 0 V, while on the other electrodes the external potential 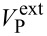 was varied from −3 V to 3 V. Ideally, the KPFM would measure the full potential difference between neighboring electrodes 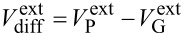. To visualize deviations form this ideal outcome, we plotted the deviation of the measured voltage 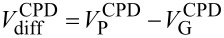 from the externally applied voltage 
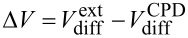
 as function of 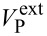 (Figure 4). Examples for crossectional potential of the interdigitated electrode array can be found in Figure S3, Figure S4, Figure S5 in Supporting Information File 1. Displayed in black is an ideal curve together with the potentials measured with the three AM-KPFM methods (red, orange and yellow) and the two FM-KPFM methods (blue and turquoise). Any positive slope in these graphs indicates that the measured potential was lower than 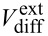. To which fraction the external voltage was captured is shown in the inset on the lower right of Figure 4. The offset in the brackets of the legend indicate the offset of the fit.

For the FM Heterodyne KPFM measurement, 99% of the potential difference was captured, which is the closest to the ideal measurement in this set of experiments. With FM Sideband KPFM 96% of the potential difference was captured. AM-KPFM on the second eigenmode captured 87% of the potential difference, which is the most accurate measurement obtained with AM-KPFM. This matches the expectation since this method utilizes the resonance enhancement. AM off resonance captured 82% of the potential difference, while AM lift mode only captured 57% of the potential difference. The huge deviation of AM lift mode could be caused by averaging. Lifting the tip up increases the contribution of the cantilever to the electrostatic force [20]. The large surface area of the cantilever leads to an averaging of the surface potential and therefore the measured potential difference is lower. The small vertical offsets in the fits could be caused by a small offset in the voltage source or by electrostatic cross talk (see discussion later in the manuscript). Nevertheless, with values *<*20 mV, these offsets are on the order of the experimental error.

**Figure 5:**
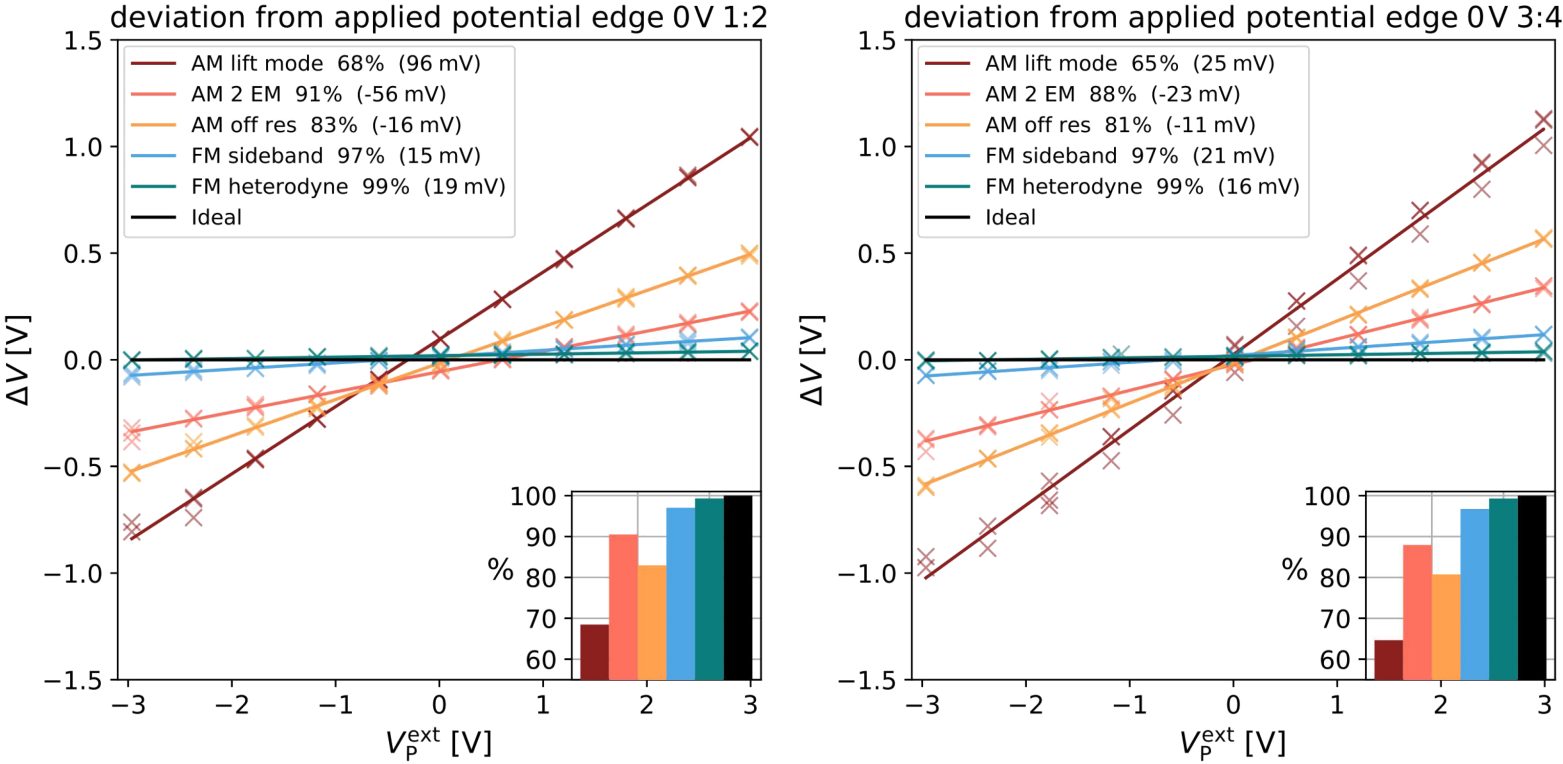
Comparison of the deviation of the measured potential difference from the applied potential plotted against the applied potential for AM (warm colors) and FM (cool colors), black represents an ideal measurement. Insert in the lower right visualizes the fraction of the potential captured by the respective method. Legend in the upper left shows the offset in brackets. Data shown captured on the electrodes 1/2 (left) and 3/4 (right) of the electrode structure without an additional electrostatic force.

The next measurement was performed close to edge of the model electrode structure at a position, where the entire length of the cantilever was over the glas substrate. This geometry is chosen to mimic the experimental situation when measuring on a device cross section. In this geometry, the cantilever is interacting with an insulating surface instead of the electrode structure. Due to this break in symmetry, we observed different results for the first two electrodes (Figure 5 left) as compared to following two electrodes (Figure 5 right).

As in the measurement in the center of the structure, the FM-KPFM methods captured more than 95% of the potential difference. The AM-KPFM modes showed a slightly better performance with AM lift mode capturing 68% of the potential difference. Nevertheless, we noted that the AM 2 EM and AM lift mode curves were vertically shifted by +56 mV and −95 mV, respectively (vertical offsets are given in brackets in the figure legends). The offsets observed with the other KPFM modes and in all measurements at the center of the device were within the experimental error. The offsets measured on the first two electrodes were larger than the offsets on the following two electrodes (Figure 5 right). The observation that this offset only appeared in AM-KPFM modes and that it was stronger closer to the edge of the structure suggests that an additional electrostatic force from the insulating substrate (i.e., a stray field) was acting on the cantilever. This electrostatic force can for example arise from static charges on the glass surface [6].

**Figure 6:**
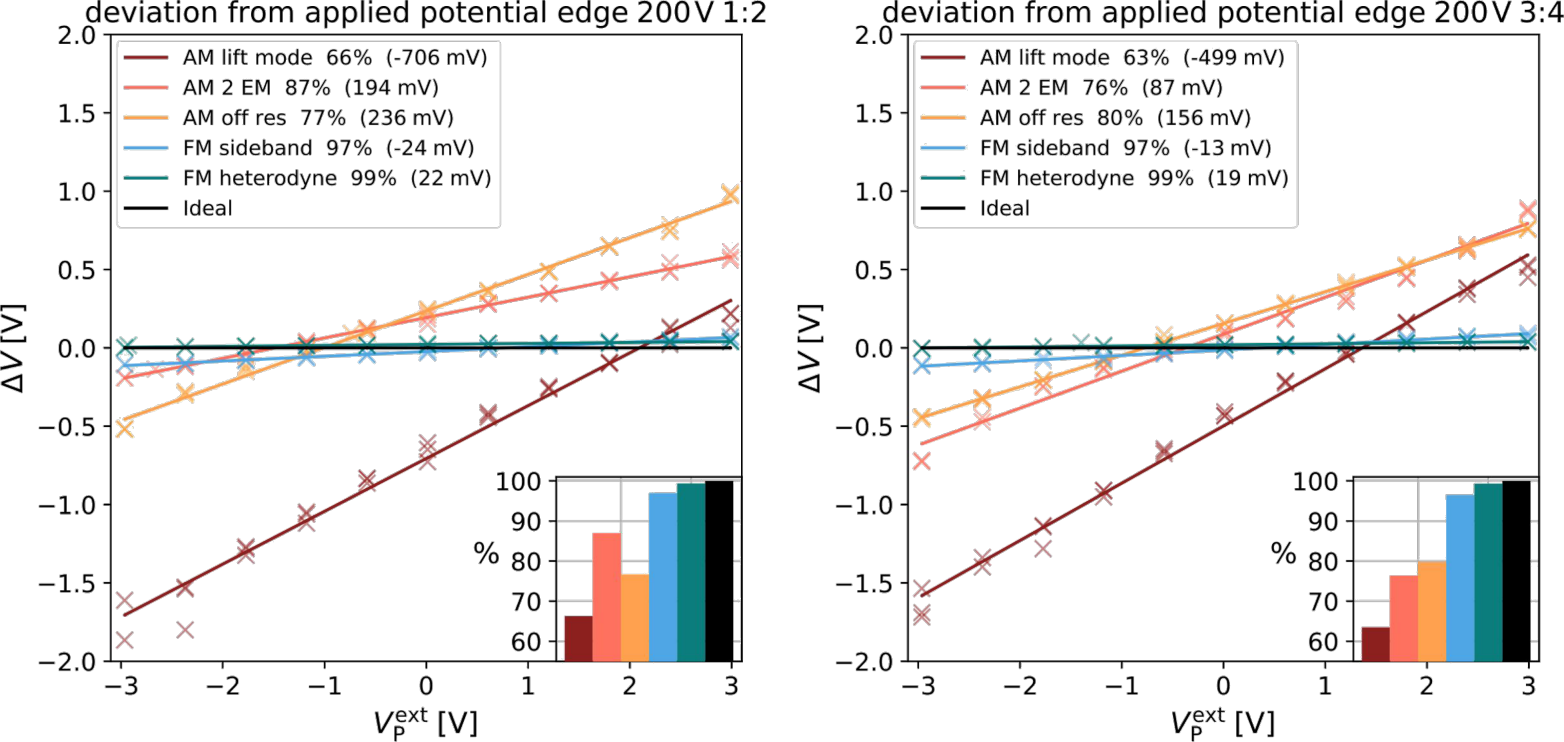
Comparison of the deviation of the measured potential difference from the applied potential plotted against the applied potential for AM (warm colors) and FM (cool colors), black represents an ideal measurement. Insert in the lower right visualizes the fraction of the potential captured by the respective method. Legend in the upper left shows the offset in brackets. Data shown captured on the electrodes 1/2 (left) and 3/4 (right) of the electrode structure with an additional electrostatic force.

To test the hypothesis of stray fields causing the offsets, we induced an artificial stray field by placing the substrate on a piece of aluminum foil and applying a voltage of 200 V with respect to the grounded electrode. We then repeated the experiments in the center and at the edge of the electrode structure. The measurement in the center of the electrode structure did not show significant changes compared to the measurement without stray field (not shown). However, the measurements at the edge of the electrode showed significant deviations (electrode 1/2: Figure 6 (left) and electrode 3/4: Figure 6 (right)). Whereas the FM modes still measured more than 97% of the applied potential with offsets of less than 25 mV, the AM measurements showed large deviations with offsets of up to 706 mV (AM lift mode). The offsets decreased, e.g., to to 500 mV in AM lift mode, on the next two electrodes 3/4 (Figure 6 (right)).

**Figure 7:**
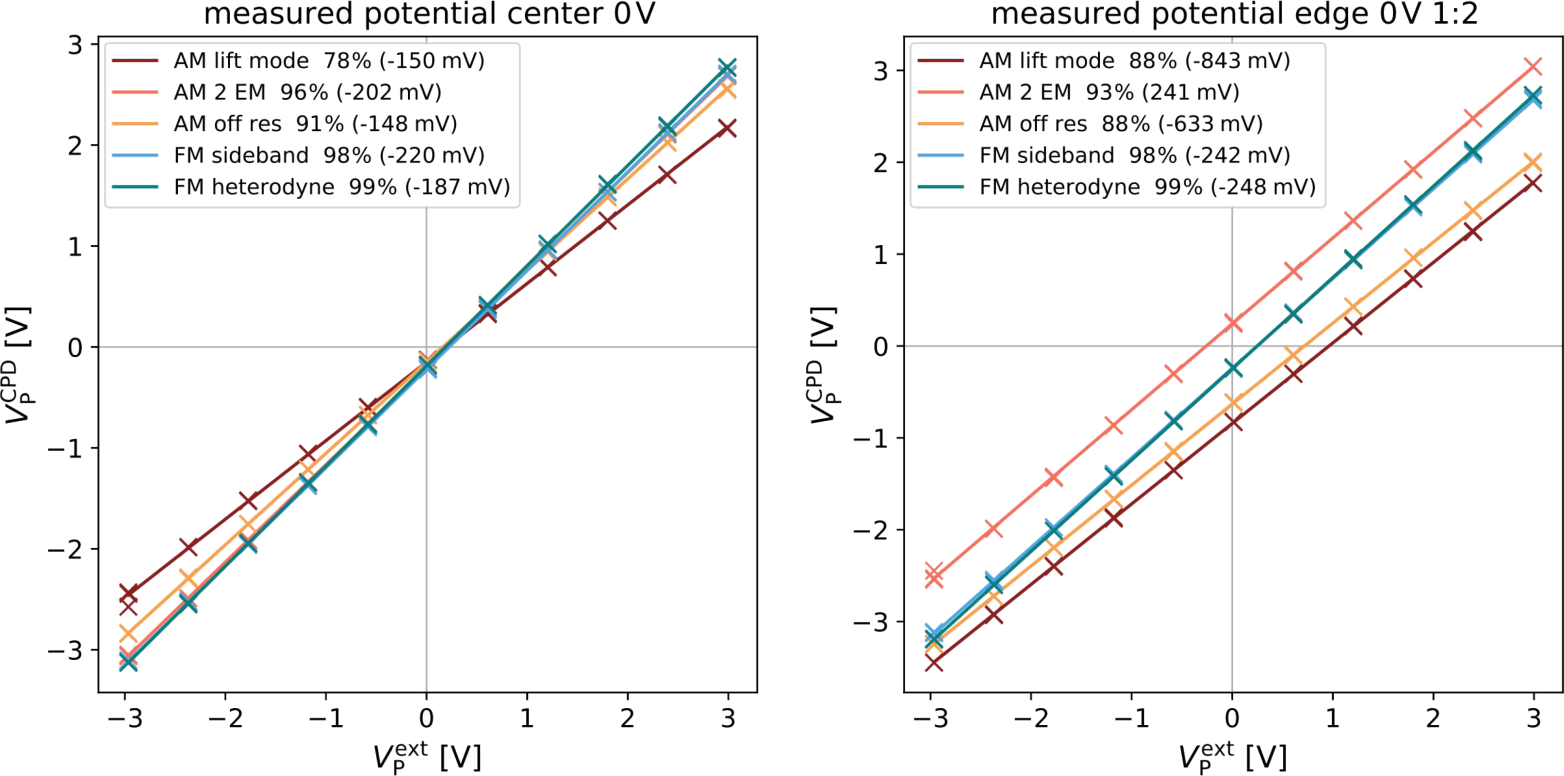
Comparison of the absolute measured potential plotted against the applied potential for AM (warm colors) and FM (cool colors). Legend in the upper left shows the measured CPD at 0 V applied voltage in brackets. Data shown captured in the middle of the electrode structure (left) and on the outer most electrodes (1/2) (right) without an additional electrostatic force.

Since a grounded reference structure is not always available or the work function of the structures is of interest, we investigated the absolute value of the measured potential, as well. The absolute measured potential on the biased electrode is plotted against the applied potential and shown for the most extreme cases: in the center of the structure and on the outer most electrodes. In the center of the structure and in the absence of a stray field, the CPD varied from −220 mV to −148 mV (Figure 7 (left)). Such variations can be due to local changes in the CPD caused by contamination of the tip or variations in the surface as well as remaining charges in the substrate surrounding the electrodes.

**Figure 8:**
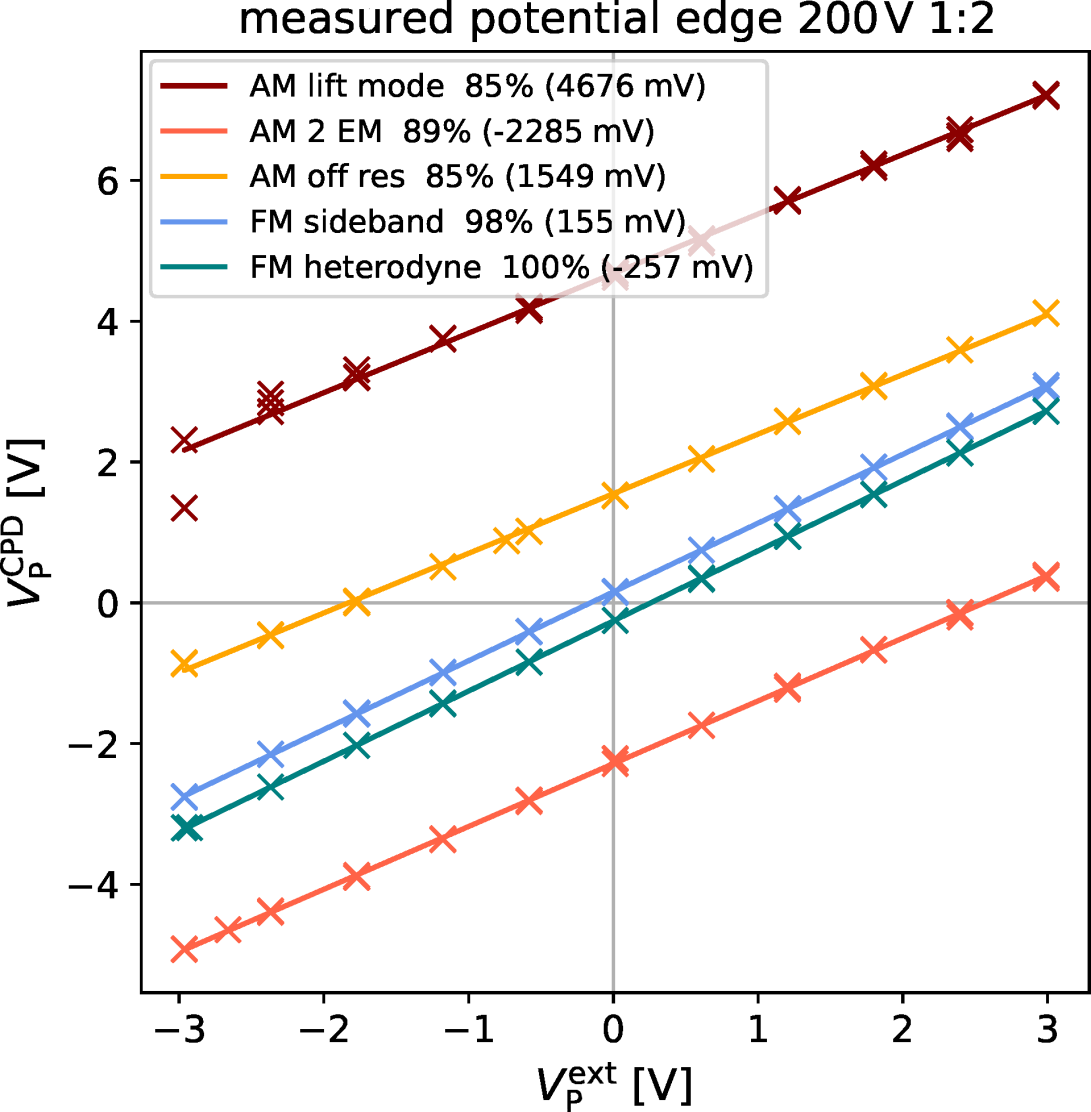
Comparison of the absolute measured potential plotted against the applied potential for AM(warm colors) and FM(cool colors). Legend in the upper left shows the measured CPD at 0 V applied voltage. Data shown captured on the outer most electrodes (1/2) of the electrode structure with an additional electrostatic force.

On the edge of the electrode structure, the measured CPD increased to up to 840 mV (Figure 7 (right)). These significant deviations are most likely enhanced by stray fields from the glass substrate. In the presence of the artificial stray field caused by the aluminum electrode underneath the substrate, the measured CPD further increased to up to 4.7 V (AM lift mode, Figure 8). Generally, the offsets in CPD were much higher in the AM-KPFM modes compared to the FM-KPFM modes, where the maximum deviation was 250 mV (FM Heterodyne). It is interesting to note that methods operating on the first eigenmode or at frequencies below exhibited a positive offset while for the detection on the second eigenmode, the offset was negative. This could be connected to the way the different motion patterns of the cantilevers fundamental and second eigenmode interact with the substrate [42]. Thereby, a position-dependent sensitivity to stray fields along the cantilever could lead to a changed overall response, depending on which eigenmode is used for the electrostatic detection. The origin of this effect could be elucidated by numerical simulations, which is beyond the scope of this work.

## Conclusion

Our results show that generally, FM-KPFM methods provide more quantitative and reliable results. For all experiments, FM-KPFM measured more than 96% of the externally applied potential difference, even in the presence of a strong stray electric field. Due to the stronger contribution of the cantilever on the measured surface potentials in AM-KPFM, the exposure of the cantilever to a stray electric field had a strong impact both on the potential difference and on the absolute potential.

With these new results, we now understand the differences in the potential distributions on the perovskite solar cell cross section that we presented in the introduction: The lower contrast in the lift mode AM-KPFM image and the shift in the absolute potential as compared to the FM sideband KPFM measurement can be explained by the stronger lateral averaging of the AM-KPFM in lift mode and the presence of a stray electric field. Such a field could originate from gallium ions deposited into the glass substrate during the focused ion beam polishing of the cross section.

Our general recommendation for quantitative device measurements is therefore to use FM-KPFM methods, not only because they gave the most accurate relative potential values but also more reliable absolute potential values. In our study, FM heterodyne KPFM [26] had the best performance, capturing 99% of the potential difference in all measurements even in the presence of strong stray fields. In addition, FM heterodyne measurements can be performed at higher detection bandwidth compared to FM sideband measurements, making the method much faster. If limited to AM methods, we recommend using resonance enhanced detection, such as AM-KPFM on the second eigenmode. AM lift mode, however, is not recommended, since it was the least quantitative method and was most strongly affected by stray electric fields. In any case, when using AM-KPFM, it is crucial to reduce the impact of stray fields and electrostatics e.g. by using a ionizing air blower. These considerations are crucial for reliable quantitative and reproducible device measurements.

## Appendix

### Connection between sideband amplitudes and force gradient

The Fourier coefficients *a_n_* for the capacitance gradient 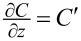 can be written as

[12]
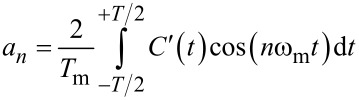


with the oscillation period 
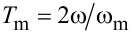
. Please note that we can use only a cosine term when we phase-shift *z*(*t*) in a way that it is symmetric around *t* = 0. Equation 12 is valid for any periodic *z*(*t*), so it is also valid for a distorted cantilever motion when the tip is interacting with the surface.

To find the connection between the Fourier coefficients and higher order capacitance derivatives, we can additionally expand *C*′ in a Taylor series around *z*_0_ = 0

[13]
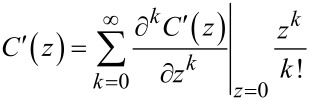


By combining the Taylor expansion until *k* = 1 with Equation 12, we can calculate the first Fourier coefficients as:

[14]
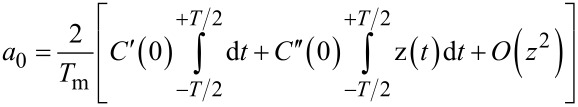


[15]
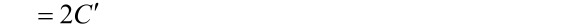


The second addend in Equation 14 becomes zero because *z*(*t*) is symmetric around *t* = 0. This proves that the AM-KPFM Equation 3 is equivalent to Equation 9. For the second Fourier coefficient we get:

[16]
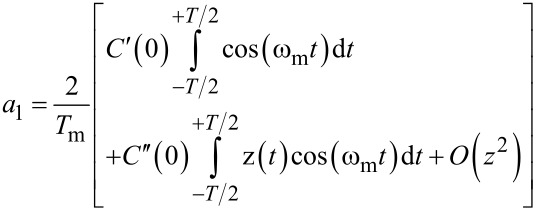


[17]
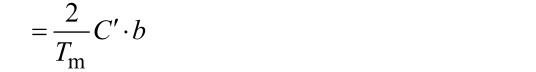


With a constant *b* ≠ 0. Here, the first addend becomes zero because cos(ω_m_*t*) is symmetric around *t* = 0. For a non-distorted cantilever motion with amplitude *A*_m_ and *z*(*t*) = *A*_m_ cos(ω_m_*t*), we obtain

[18]
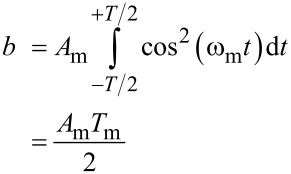


[19]
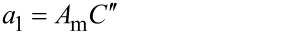


Thus, the sideband Equation 9 becomes:

[11]
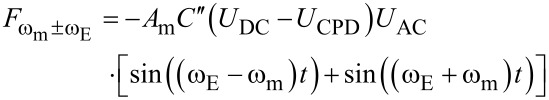


Thus, the sideband amplitude is proportional to the second derivative of the tip/cantilever-sample capacitance and the carrier amplitude *A*_m_.

## Supporting Information

File 1 Additional figures.
