# Supplementary material for: Know your full potential: Quantitative Kelvin probe force microscopy on nanoscale electrical devices
Source: Beilstein J Nanotechnol. 2018 Jun 15;9:1809–19. doi: 10.3762/bjnano.9.172 (PMC6009372; doi:10.3762/bjnano.9.172)
Supplement: File 1 — Additional figures. [file Beilstein_J_Nanotechnol-09-1809-s001.pdf]

Supporting Information

for

Know your full potential: Quantitative Kelvin probe force microscopy on nanoscale electrical devices

Amelie Axt[1,2], Ilka M. Hermes[1], Victor W. Bergmann[1], Niklas Tausendpfund[2] and Stefan A. L. Weber*[1,2]

Address: [1]Max-Planck-Institute for Polymer Research, Ackermannweg 10, 55128 Mainz, Germany and [2]Institute of Physics, Johannes Gutenberg University Mainz, 55099 Mainz, Germany

Email: Stefan A. L. Weber - webers@mpip-mainz.mpg.de

* Corresponding author

Additional Figures

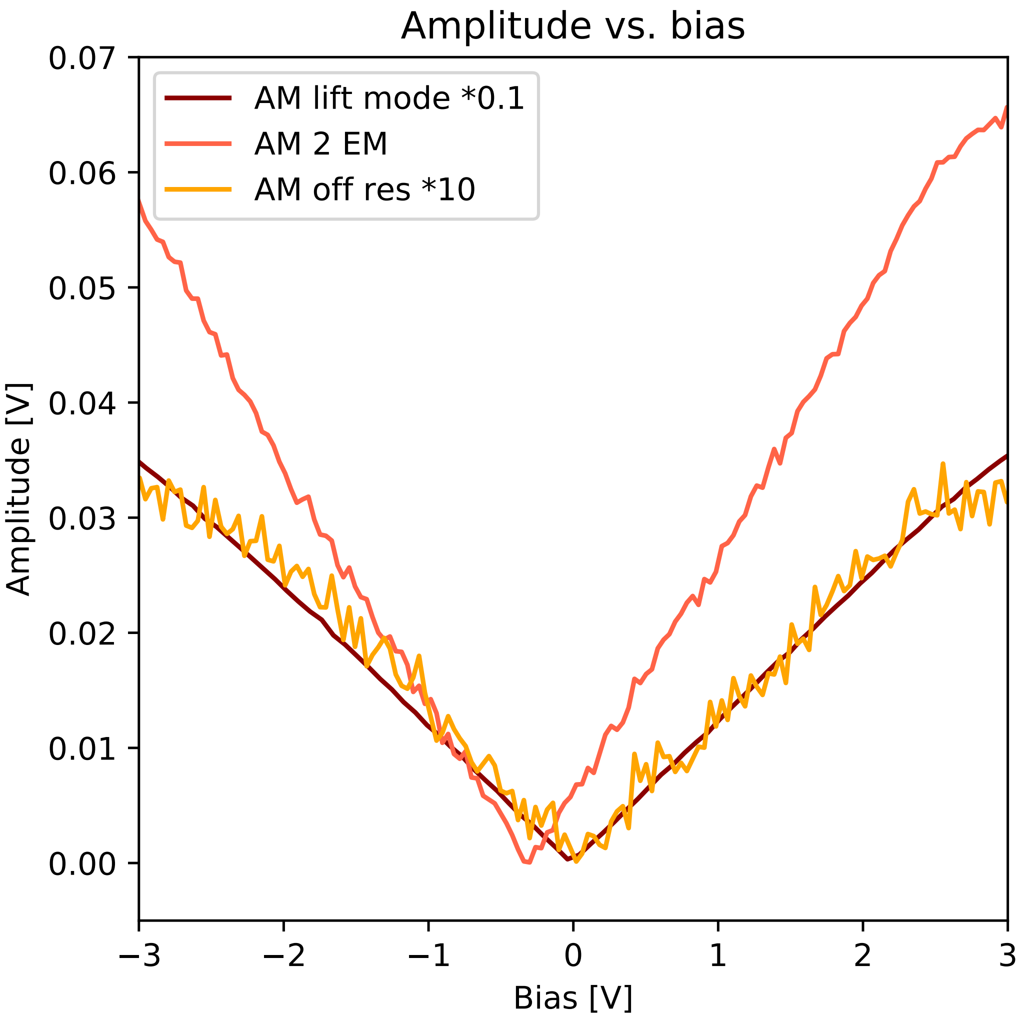

Figure S1: Amplitude vs Bias voltage applied to the tip. Numbers in the legend indicate for the multiplication factor used for plotting.

The data plotted in figure S1 and figureS2 is obtained prior to the respective KPFM images. The bias voltage is applied to the tip. In figure S1 AM 2 EM and Am off res show a slight bend downwards at −3 V and 3 V. This is most likely due to the AFM feedback, which retracts due to the strong electrostatic force. The same behaviour can be observed in figure S2 for FM sideband already starting from −1.5 V and 1.5 V. We do not expect this to have an influence on our potential measurements, since during the scans the KPFM feedback minimized the electrostatic force. The difference between the voltage applied to the tip and the CPD therefor was in the linear regime for all KPFM methods at all times.

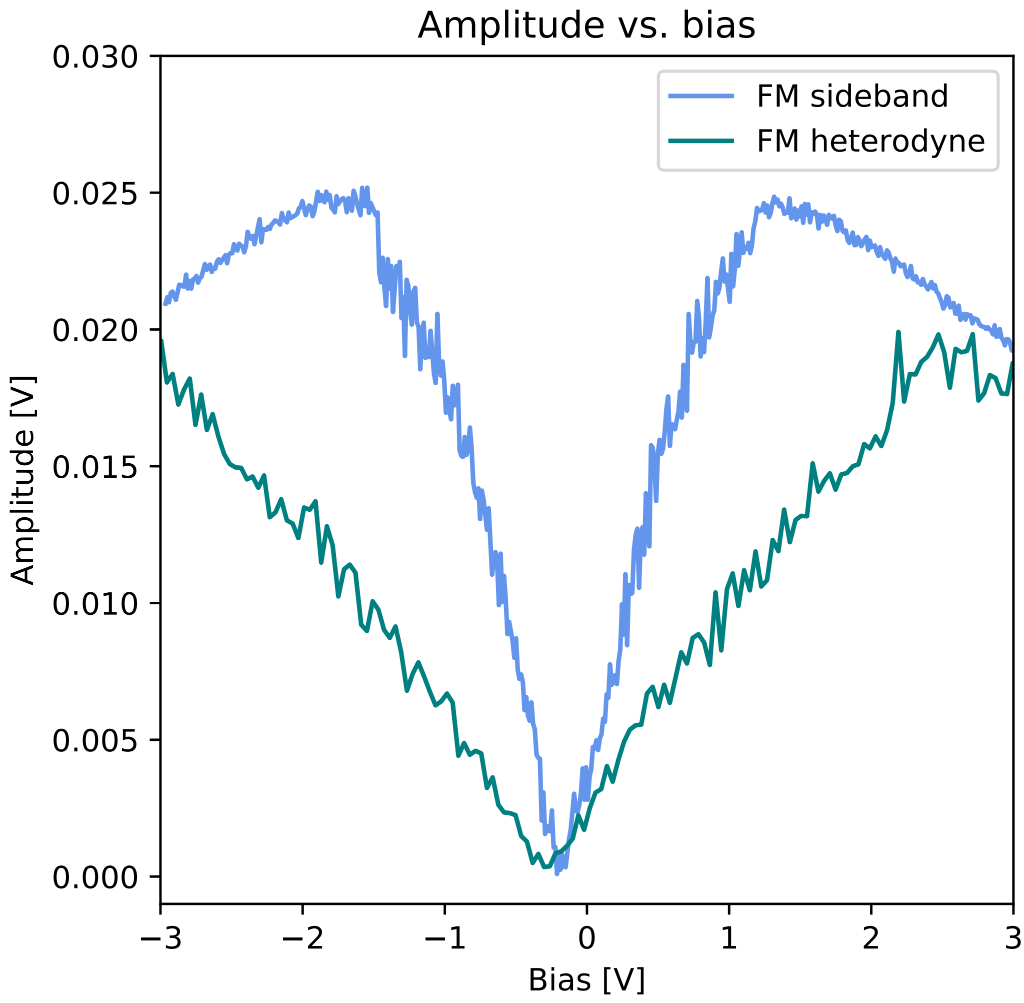

Figure S2: Amplitude vs Bias voltage applied to the tip.

Data presented in the crossectional plots was recorded in a subsequent measurement with higher resolution and therefor higher sampling rate. The absolute measured potential differs from the data above.

The measured on the elektrode on which the potential was varied on plotted against the externally applied voltage indicates crosstalk originating from uncompensated electrostatic interaction. The asymmetry in the feedback could originate from the feedback being optimized for $V_P^{ext} = 0.5$ V. The feedback therefor works better for positive V_P^{ext}. The parabolic shape is likely due to the quadratic dependence of the electrostatic force on the potential difference (Equation 1 of the main manuscript).

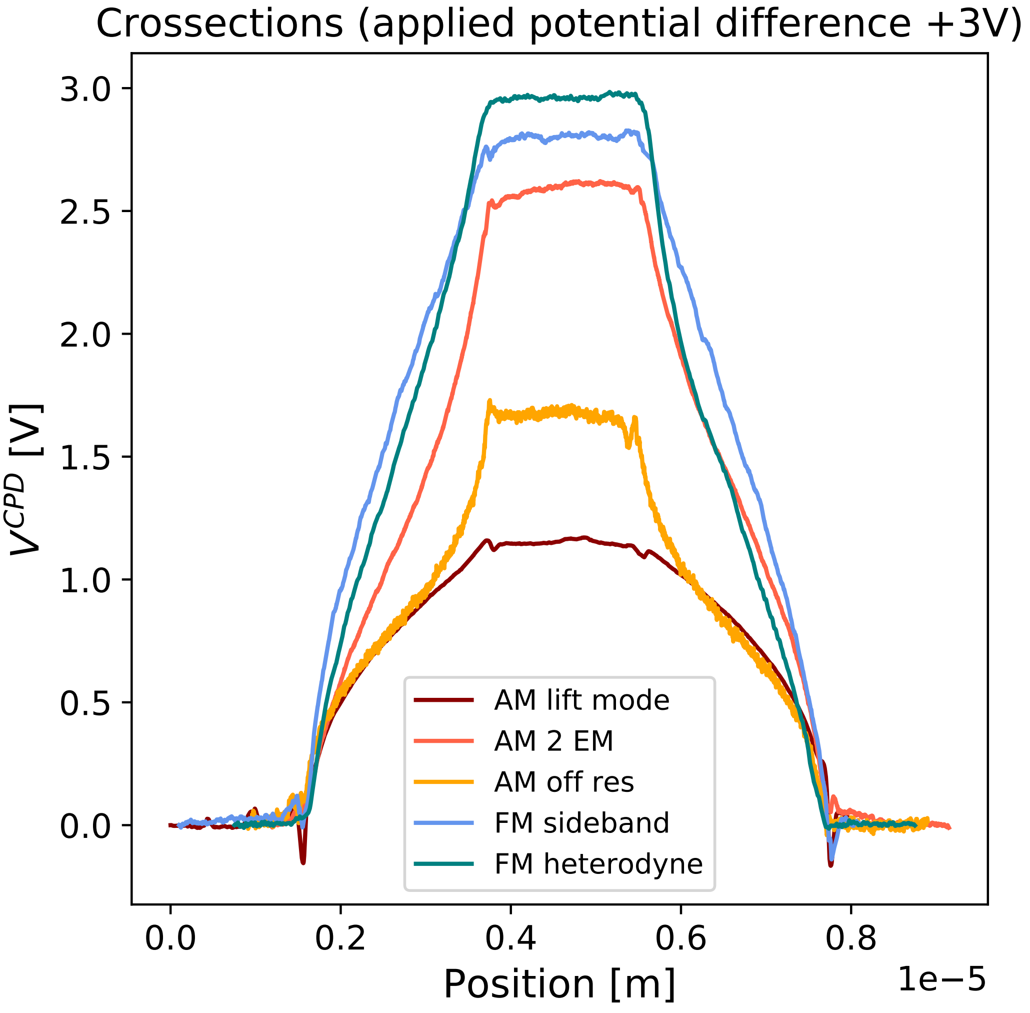

Figure S3: Comparison of the measured potential plotted against position along the *x*-axis (perpendicular to the electrode structure) for AM(warm colors) and FM(cool colors). Data shown captured in the center of the structure without application of an additional electrostatic force. The potential difference was +3 V. All crossections are normalized to start at 0 V.

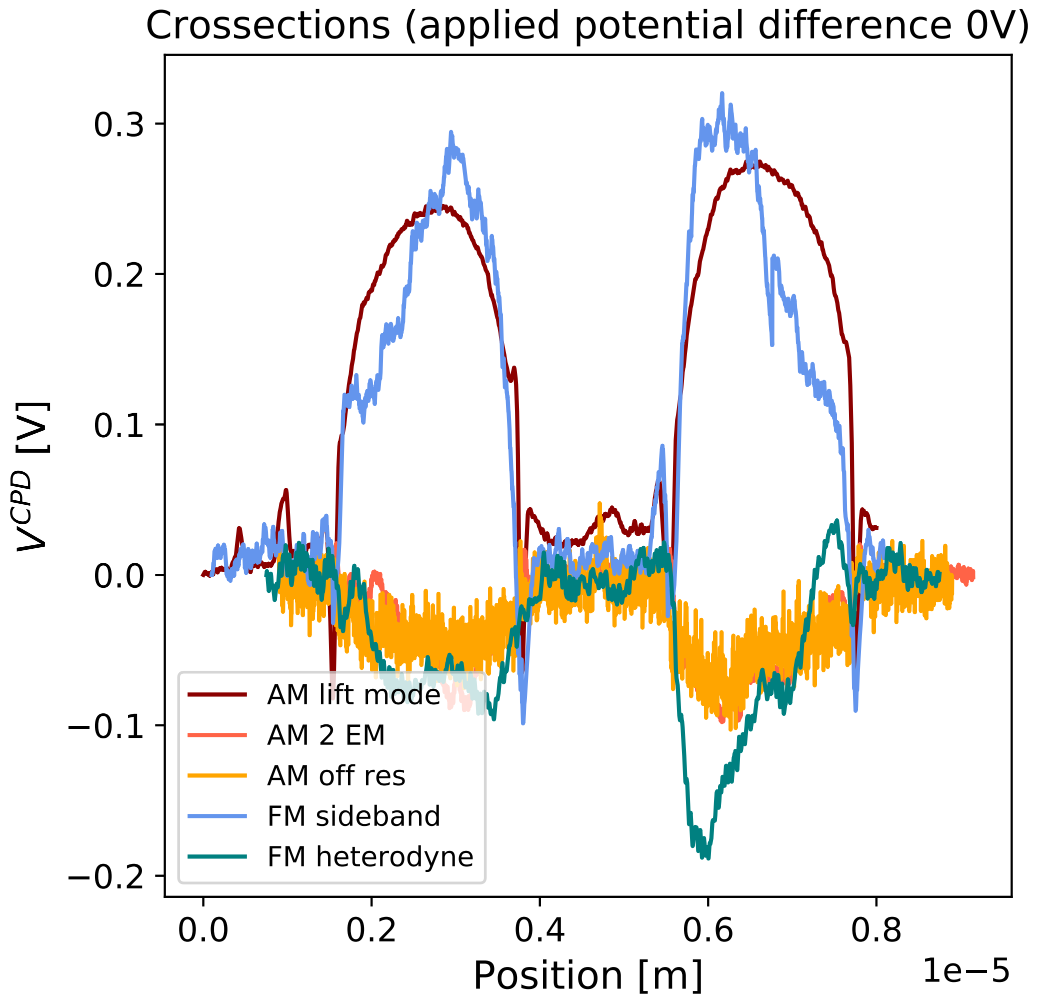

Figure S4: Comparison of the absolute measured potential plotted against position along the x-axis (perpendicular to the electrode structure) for AM (warm colors) and FM (cool colors). Data shown captured in the center of the structure without application of an additional electrostatic force. The potential difference was 0 V. All crossections are normalized to start at 0 V.

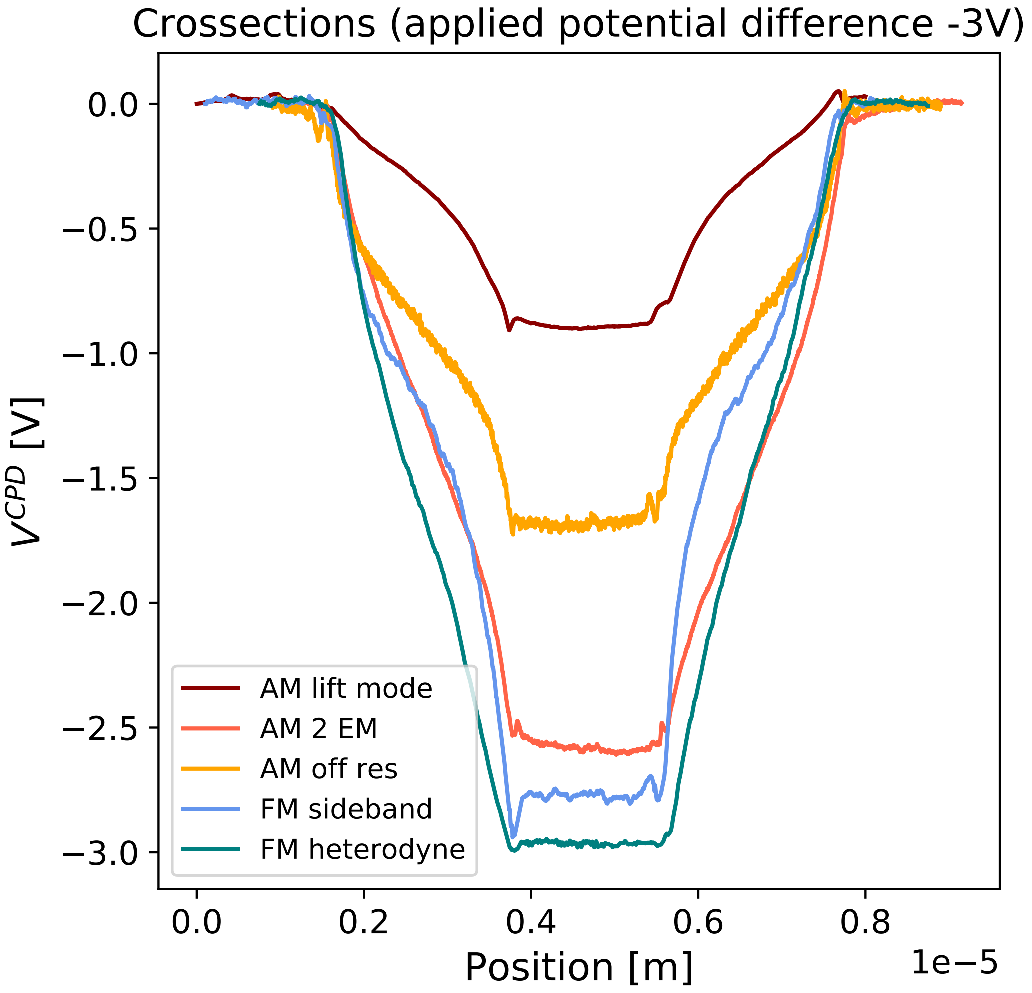

Figure S5: Comparison of the absolute measured potential plotted against position along the *x*-axis (perpendicular to the electrode structure) for AM(warm colors) and FM(cool colors). Data shown captured in the center of the structure without application of an additional electrostatic force. The potential difference was −3 V. All crossections are normalized to start at 0 V.

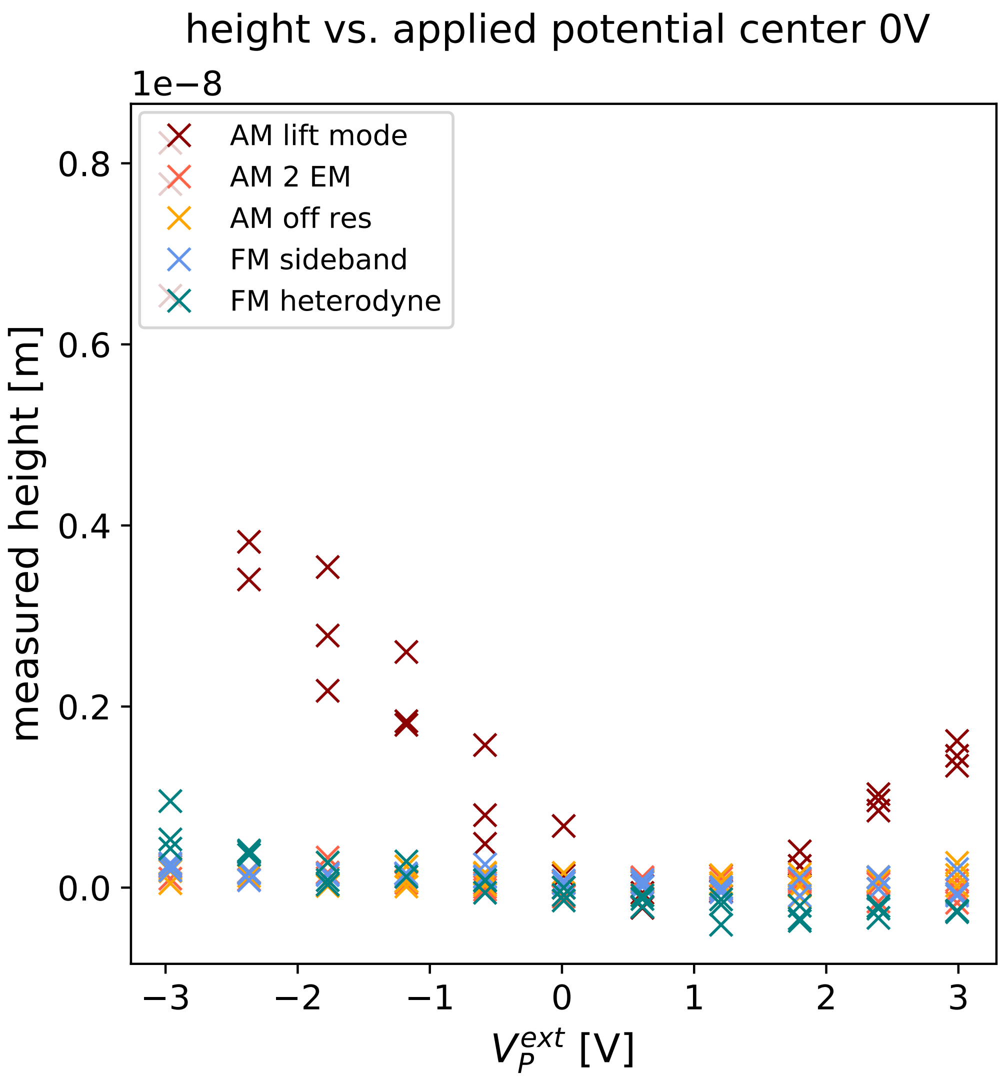

Figure S6: The height measured on the electrode on which the potential was varied plotted against the externally applied potential.

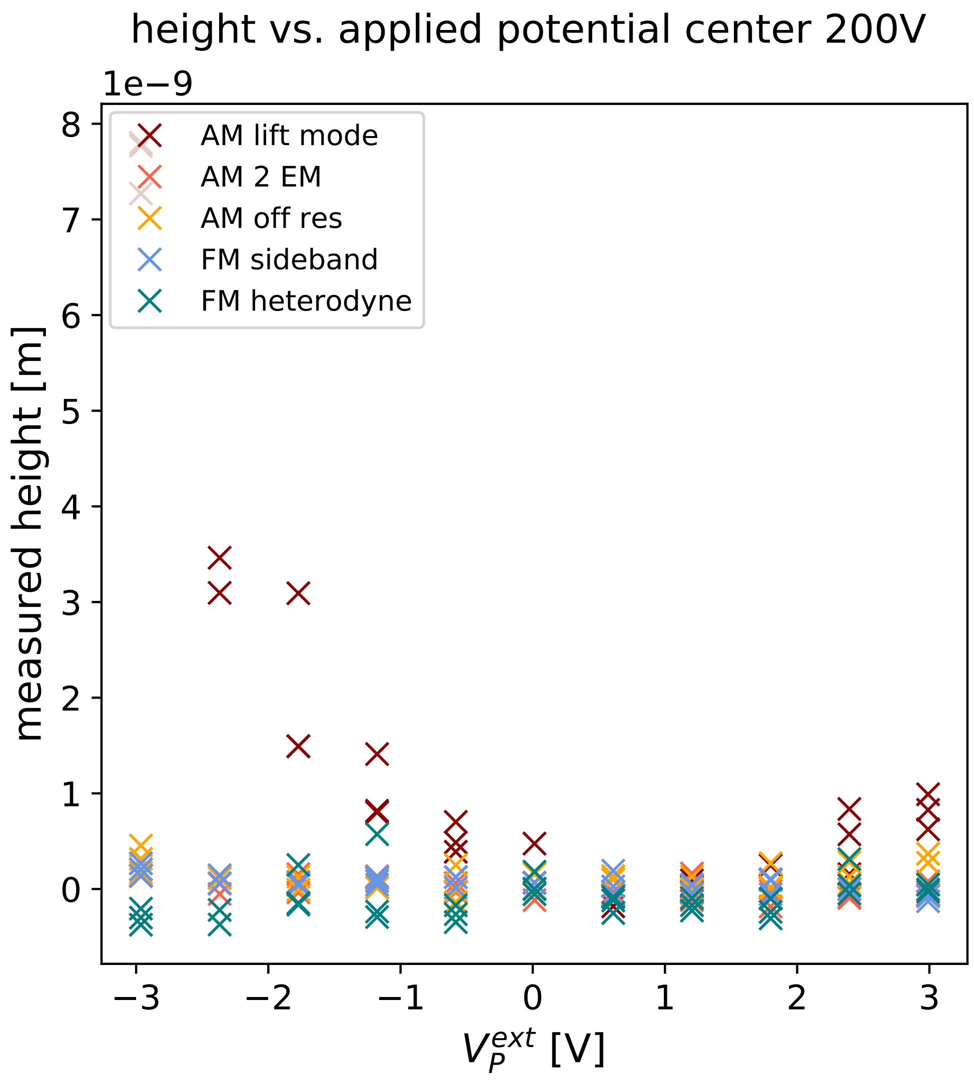

Figure S7: The height measured on the electrode on which the potential was varied plotted against the externally applied potential.

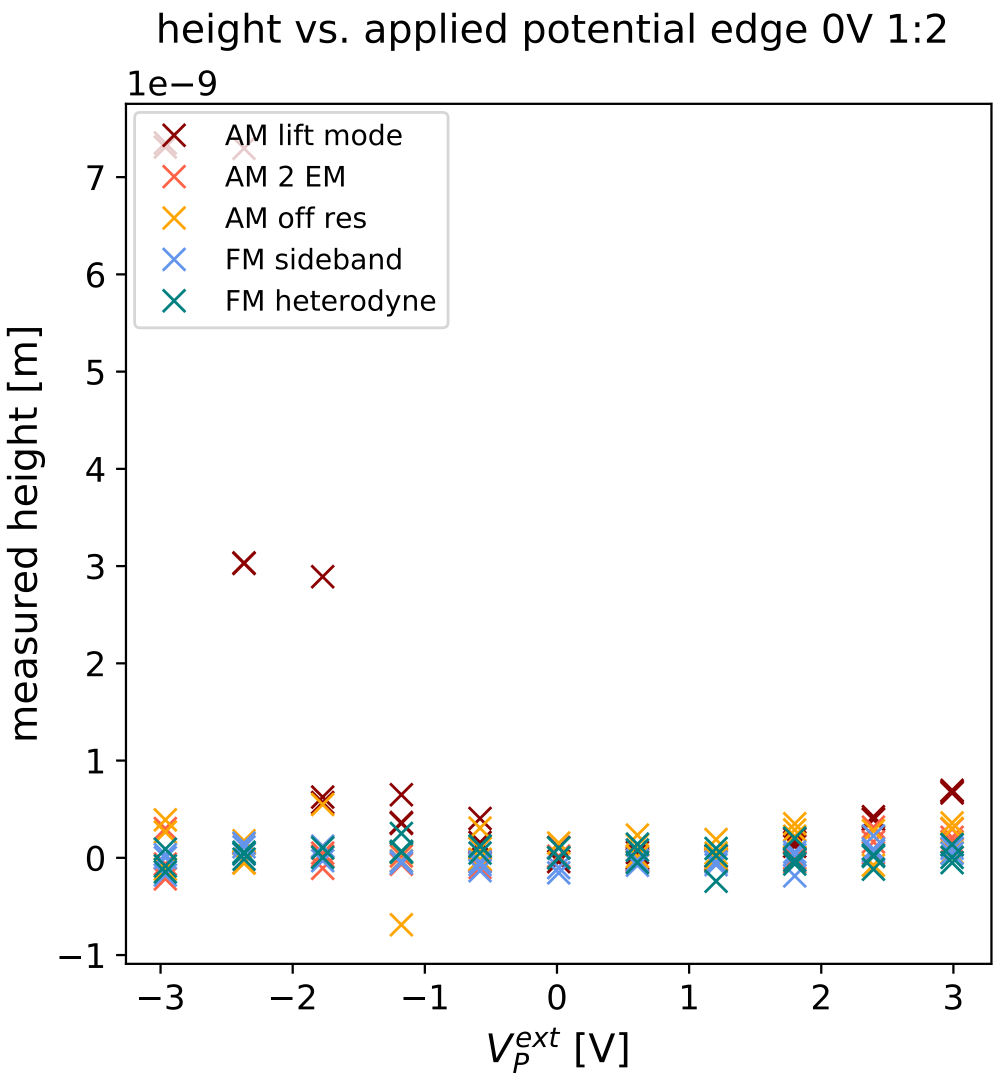

Figure S8: The height measured on the electrode on which the potential was varied plotted against the externally applied potential.

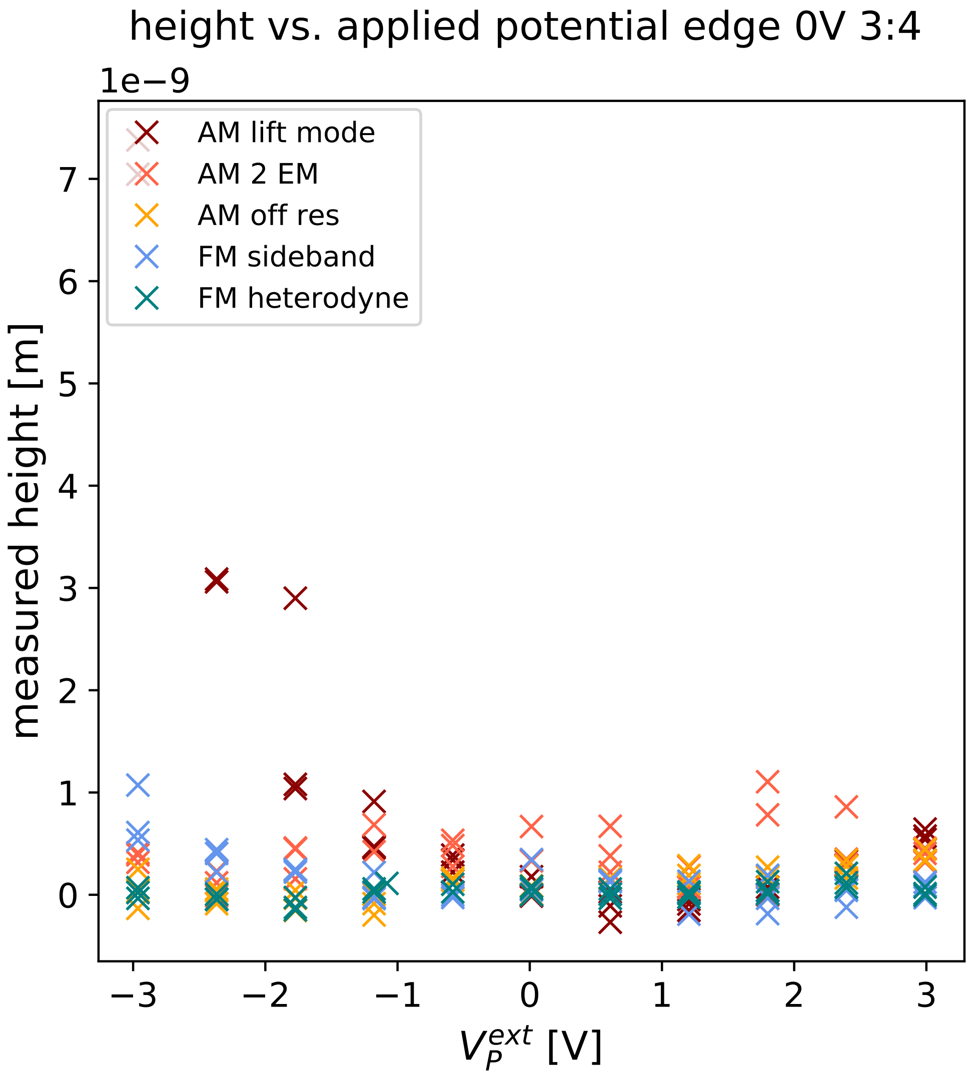

Figure S9: The height measured on the electrode on which the potential was varied plotted against the externally applied potential.

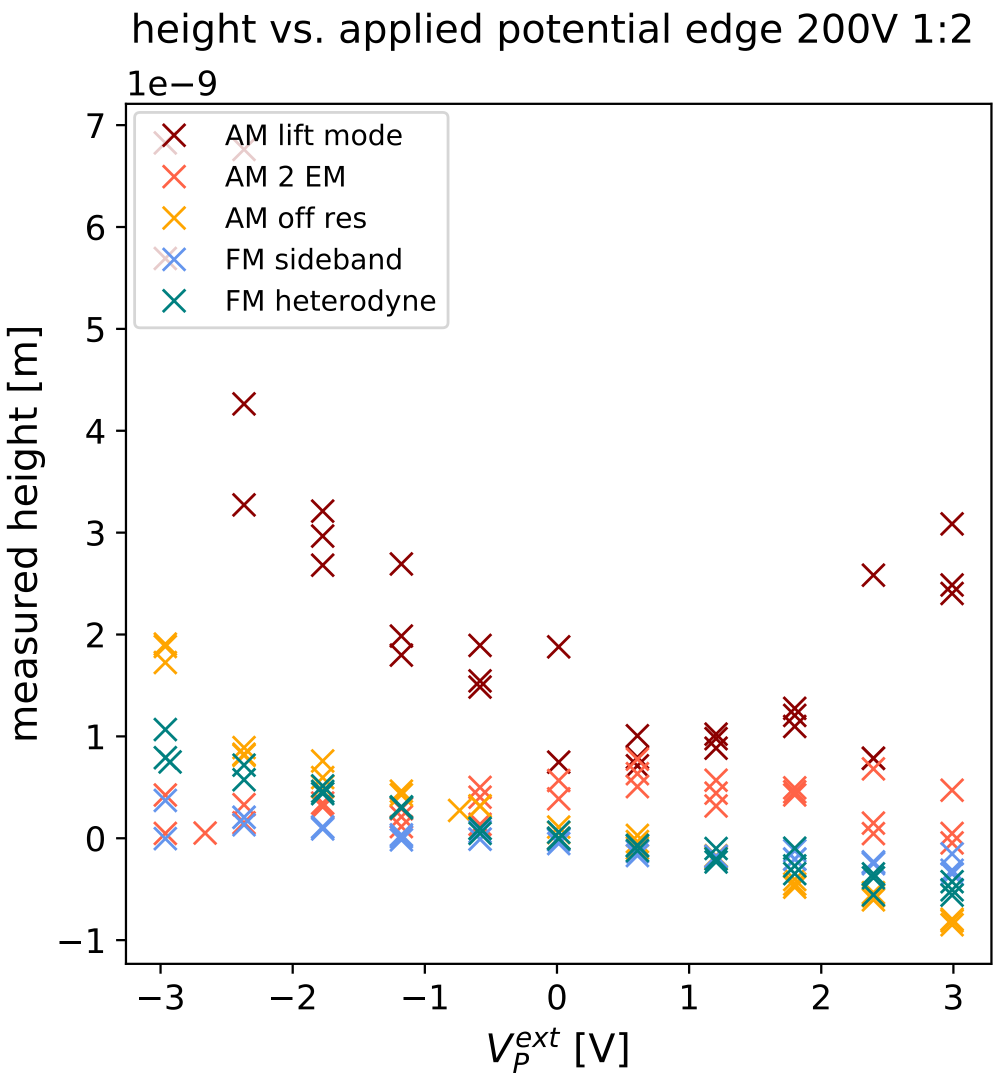

Figure S10: The height measured on the electrode on which the potential was varied plotted against the externally applied potential.

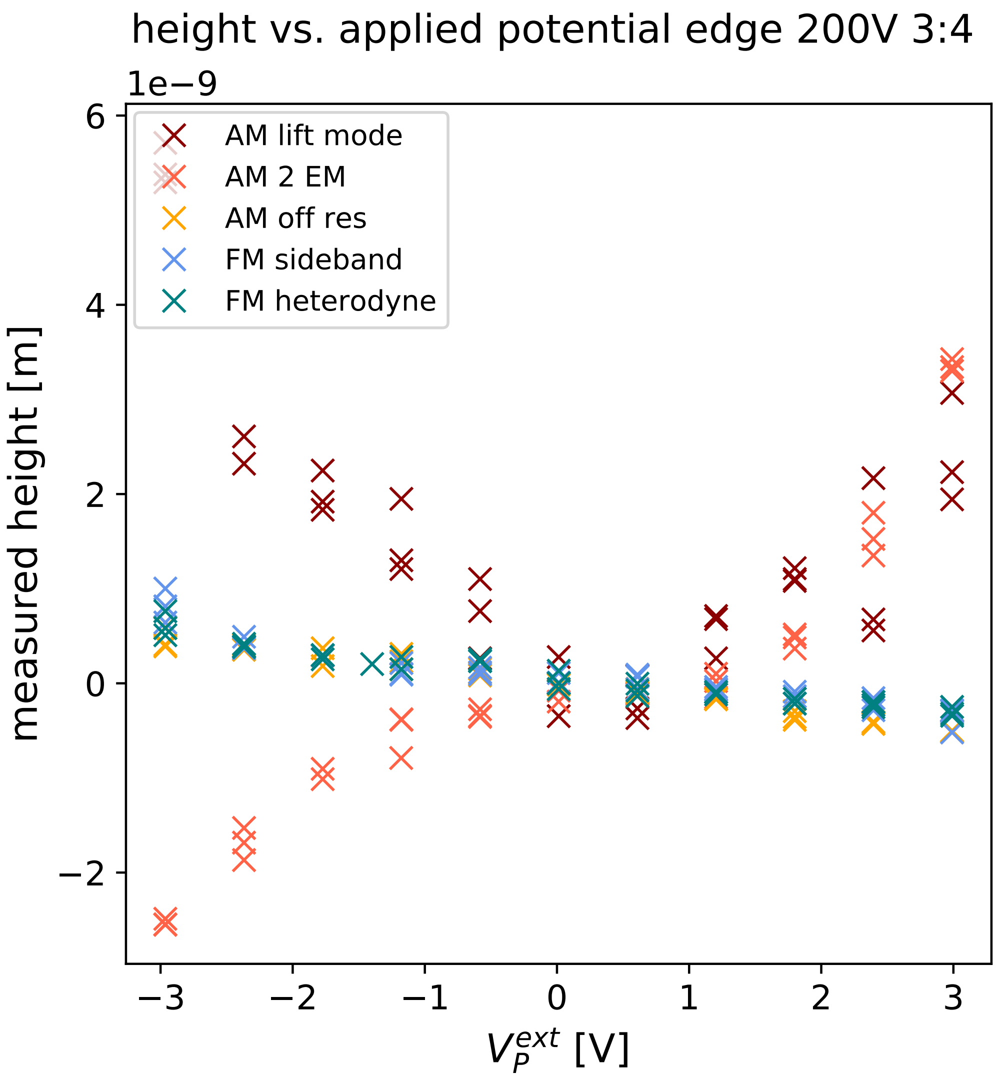

Figure S11: The height measured on the electrode on which the potential was varied plotted against the externally applied potential.

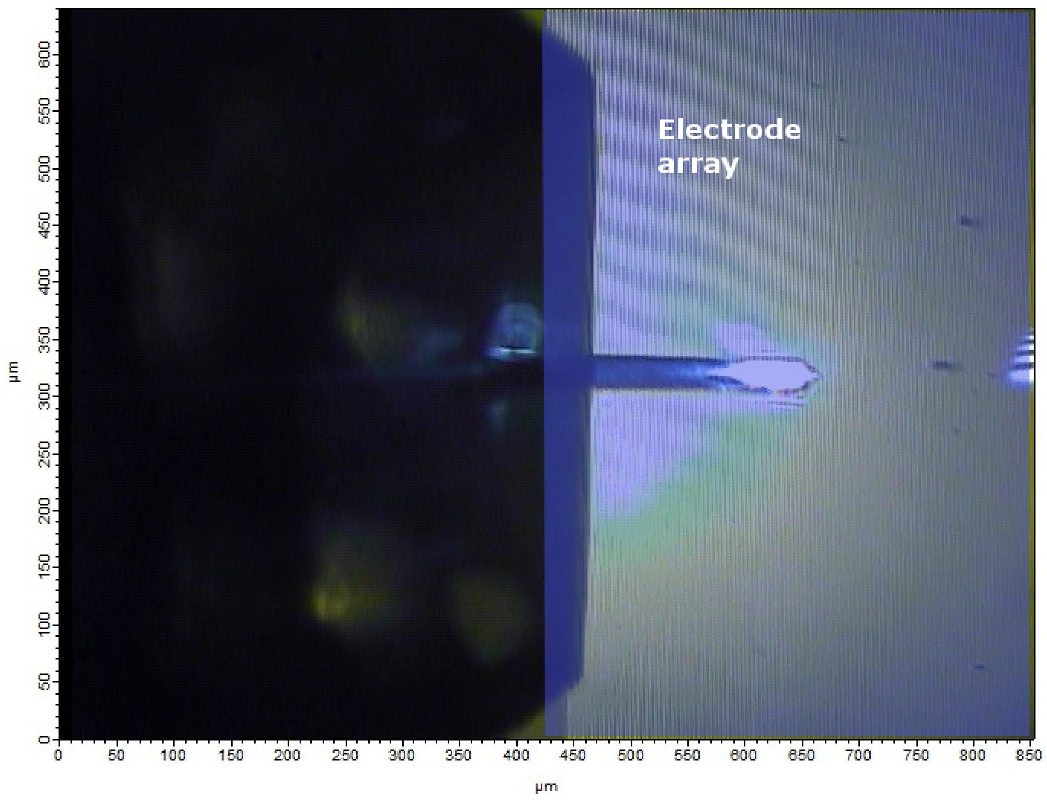

Figure S12: Positioning of the cantilever with respect to the electrode array in the center.

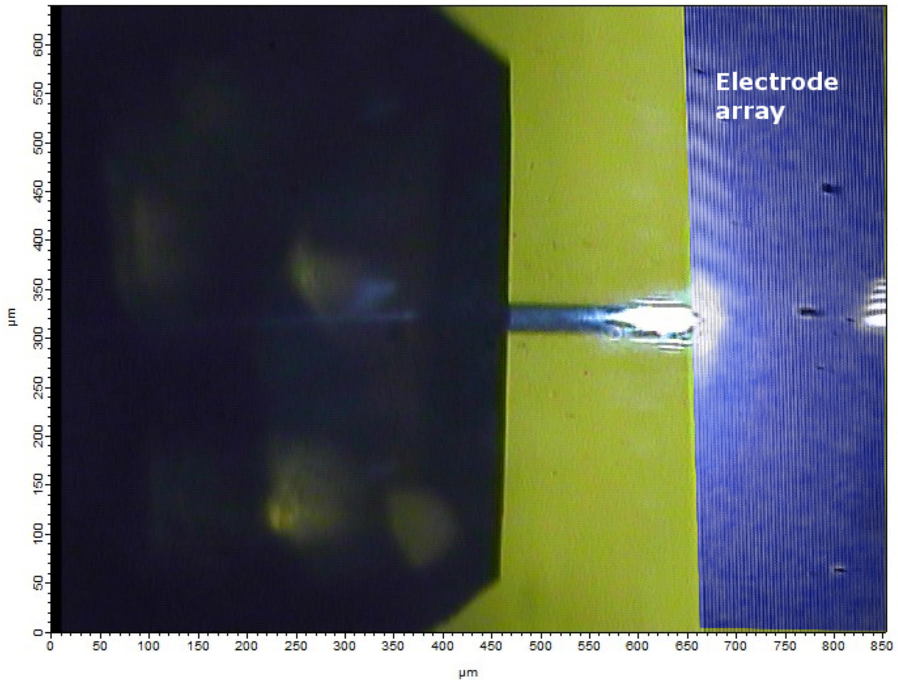

Figure S13: Positioning of the cantilever with respect to the electrode array on the edge of the array.

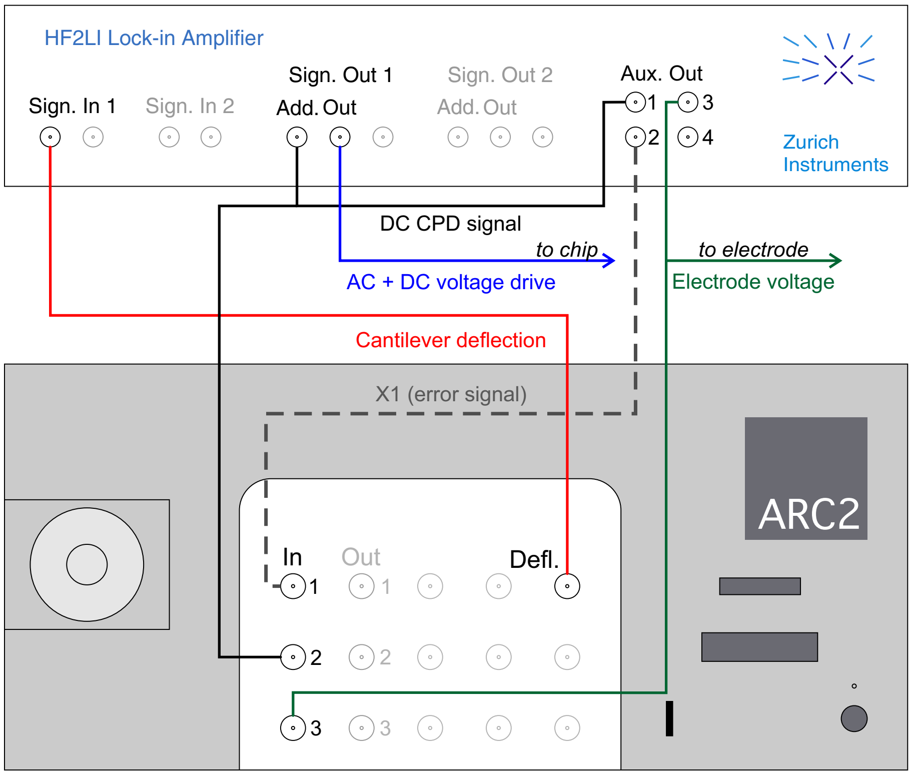

Figure S14: Circuit diagram for AM methods.

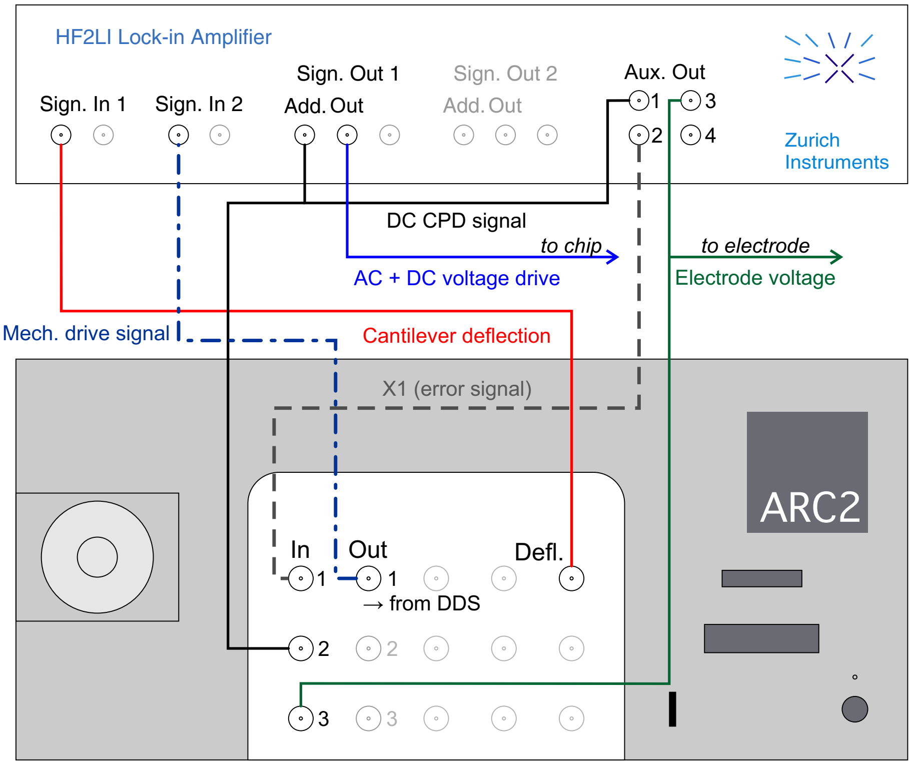

Figure S15: Circuit diagram for FM methods..
